# Mating Disruption of *Pseudococcus calceolariae* (Maskell) (Hemiptera, Pseudococcidae) in Fruit Crops

**DOI:** 10.3390/insects12040343

**Published:** 2021-04-13

**Authors:** Carolina Ballesteros, Alda Romero, María Colomba Castro, Sofía Miranda, Jan Bergmann, Tania Zaviezo

**Affiliations:** 1 Facultad de Agronomía e Ingeniería Forestal, Pontificia Universidad Católica de Chile, Avda. Vicuña Mackenna 4860, Macul, Santiago 7820436, Chile; cballestero@uc.cl (C.B.); aromeroga@uc.cl (A.R.); mccastro3@uc.cl (M.C.C.); smiranda1@uc.cl (S.M.); 2 Instituto de Química, Pontificia Universidad Católica de Valparaíso, Avda. Universidad 330, Curauma, Valparaíso 2340000, Chile; jan.bergmann@pucv.cl

**Keywords:** citrophilous mealybug, IPM, semiochemicals, sex pheromones, sustainable pest control

## Abstract

**Simple Summary:**

The citrophilous mealybug is an economically important pest that is mainly controlled using insecticides, not always successfully, and with unintended negative environmental side effects. In our research, we tested a specific and sustainable control tool using the mealybug sex pheromone. Mating disruption is a technique that aims to reduce mating between males and females by inundating the area with the synthetic sex pheromone of the species, thereby reducing reproduction and consequently populations over time and damage. For this purpose, the mealybug pheromone, incorporated into a polymeric substance for its release, was applied in a tangerine and an apple orchard, in two seasons (2017/2018 and 2019/2020). In all seasons, a reduction in the males catches in traps after deploying pheromone was observed, which would indicate a decrease in the probability of successful mating compared to control plots. The duration of this effect was around one year. Mealybug abundance on trees was extremely low throughout the trials, so it was not possible to observe a reduction of populations or damage. This research shows that the use of this pheromone-based technique has good potential for controlling the citrophilous mealybug, with the advantage of being environmentally friendly and non-toxic.

**Abstract:**

*Pseudococcus calceolariae*, the citrophilous mealybug, is a species of economic importance. Mating disruption (MD) is a potential control tool. During 2017–2020, trials were conducted to evaluate the potential of *P. calceolariae* MD in an apple and a tangerine orchard. Two pheromone doses, 6.32 g/ha (2017–2018) and 9.45 g/ha (2019–2020), were tested. The intermediate season (2018–2019) was evaluated without pheromone renewal to study the persistence of the pheromone effect. Male captures in pheromone traps, mealybug population/plant, percentage of infested fruit at harvest and mating disruption index (MDI) were recorded regularly. In both orchards, in the first season, male captures were significantly lower in MD plots compared to control plots, with an MDI > 94% in the first month after pheromone deployment. During the second season, significantly lower male captures in MD plots were still observed, with an average MDI of 80%. At the third season, male captures were again significant lower in MD than control plots shortly after pheromone applications. In both orchards, population by visual inspection and infested fruits were very low, without differences between MD and control plots. These results show the potential use of mating disruption for the control of *P. calceolariae.*

## 1. Introduction

Pheromones and other semiochemicals have been used in pest management worldwide for more than 50 years, becoming an important tool in the development of sustainable management strategies for agriculture and forest pests [1]. Their use in pest management can be for detection, monitoring or control [1,2,3,4,5]. Their use for pest control may be through mass trapping, “attract and kill”, or mating disruption (MD). MD has been one of the most successfully used strategies for controlling various pests, with over 800,000 hectares treated worldwide [1,2,3,6]. MD has been mainly used for the control of Lepidoptera species attacking vegetables, orchards and forests [7], such as codling moth (*Cydia pomonella* L.), grapevine moth (*Lobesia botrana* Denis & Schiffermüller), the plum fruit moth (*Grapholita funebrana* Treitschke) [8] and gypsy moth (*Lymantria dispar* L.) [1,2,6,9]. Successful cases of mating disruption have also been reported in other insect orders such as the Oriental beetle (*Anomala orientalis* (Waterhouse)) and *Prionus californicus* Motschulsky, both Coleoptera [2,10,11,12]. Only two commercial formulations of MD have been developed so far for Hemiptera, one against the California red scale, *Aonidiella aurantii* (Maskell) (Diaspididae), the other for the vine mealybug, *Planococcus ficus* Signoret (Pseudococcidae) [3,13,14,15].

Although the first pheromones for Pseudococcidae were identified in the early 1980s from *Pseudococcus comstocki* (Kuwana) [16] and *Planococcus citri* (Risso) [17], the identification and synthesis of pheromones of new species resumed only from 2001 onwards, summing to date 21 species [17,18,19,20,21,22,23,24,25,26,27,28,29,30,31,32]. The most studied mealybug species worldwide has been *Pl. ficus*, given its great economic importance as a key pest in several crops, particularly grapes. Its sex pheromone was first described by Hinkens et al. [18], and later monitoring methods [33,34] and mating disruption studies were developed [15,34,35,36,37,38]; it is the first mealybug species for which the successful use of MD has been reported. An additional case of successful application of MD for a mealybug species has been reported for *Planococcus kraunhiae* (Kuwana) in Japanese persimmon orchards [39].

*Pseudococcus calceolariae* (Maskell), commonly known as citrophilous mealybug, is distributed in Australia, New Zealand, USA, South Africa, and several countries in South America and south-eastern Europe [40,41]. This mealybug is a polyphagous species attacking several fruit crops such as orange, lemon, apple, pear, and avocado. Its main economic impact is due to its quarantine status for many markets. Usually synthetic insecticides are used for its control, although with low effectiveness due to its cryptic habit [38,42]. The sex pheromone of *P. calceolariae* was identified as (1*R*,3*R*)-chrysanthemyl (*R*)-2-acetoxy-3-methylbutanoate [27,28,43]. The potential use of the *P. calceolariae* pheromone for monitoring its presence and abundance in fruit crops has been reported [43,44]. Recently, Sullivan et al. [41] studied the potential use of the pheromone for the control of *P. calceolariae* through a mass trapping approach, showing a 90% decrease in male catches in treated plots. The objectives of the present study were to determine the potential for using *P. calceolariae* pheromone for control through a mating disruption (MD) approach in fruit orchards and to study the persistence of the disruption effect under field conditions.

## 2. Materials and Methods

### 2.1. Experimental Sites and Treatment Applications

Field trials were conducted in two commercial orchards in central Chile: a conventionally managed tangerine orchard (6 years-old, cv. Orri, planted at 7 × 2 m) located near Pomaire (−33.64° S, −71.13° W) in the Metropolitan Region, and an organic apple orchard (10 years-old, cv. Fuji, planted at 2.7 × 2 m) near San Fernando (−34.59° S, −70.98° W) in the O’Higgins Region. Both orchards were infested with *P. calceolariae*. In September 2017, 10 plots of 0.1 ha each (experimental unit), separated by at least 50 m, were selected in each orchard. Five plots of these units were randomly selected to receive the mating disruption pheromone treatment (MD) and the other half were used as controls (Control). An isomeric mixture of chrysanthemyl 2-acetoxy-3-methylbutanoate was synthesized as described previously in El-Sayed et al. [27]. The proportion of the active isomer (*R*, *R*, *R*) in the mix was approximately 15%. Previous studies have shown that the non-natural isomers do not interfere with male attraction [43]. On 19–20 December 2017 the pheromone was applied in the tangerine and apple orchards using SPLAT^®^ (Specialized Pheromone & Lure Application Technology; ISCA Technologies, Riverside, CA, USA) at a dose of 6.32 g/ha of isomeric mixture. This dose was based on a small-scale study that we carried out in 2015. SPLAT^®^ is an emulsified microcrystalline wax matrix allowing controlled release of formulated volatile compounds [45] that has been successfully used for MD in lepidopteran and coleopteran pests [45,46,47,48,49,50]. It can be applied manually or mechanically to the crop, it is biodegradable and has a low production cost [45]. This matrix (1 g approx.) was placed on a piece of cardboard of 20 cm^2^ that was hung on trees, with a density of 750 dispensers / ha (i.e., 75 in the 0.1 ha plots). Control plots received only SPLAT^®^.

To determine mating disruption effect over time, treatments were not reapplied in the 2018–2019 season, but dispensers were left hanging on the trees. In September 2019, treatments were deployed again in the same plots; three plots were relocated in the apple orchard because of very low populations. The pheromone dose was increased to 9.45 g/ha in this field season because it was deployed earlier in the season, and also because similar studies with *Pl. ficus* have used higher doses. Moreover, in this season the SPLAT^®^ application was made directly to the tree branches in tangerines, to avoid the cardboard being broken and/or removed due to pruning.

### 2.2. Male Captures in Pheromone Traps

To determine if a mating disruption effect was occurring, males were monitored by placing one Delta sticky trap in the center of each 0.1 ha plot (Feromonas Chile LTDA, Santiago, Chile) with rubber septum lures loaded with 50 µg (isomeric mixture) of *P. calceolariae* sex pheromone. Traps were deployed in September 2017, 3 months before the first pheromone application, and from then on continuously monitored until March 2020 (30 months, 50–52 monitoring occasions). This comprised three entire apple productive seasons (September–March in the years 2017/2018; 2018/2019 and 2019/2020) and two and a partial third for tangerine (September–August 2017/2018 and 2018/2019, and September–March 2019/2020). The sticky floors of the traps were replaced every two weeks from August to April (spring-summer) of each year and monthly from May to July (autumn-winter) of each year. Lures were renewed in July 2018 and August 2019 in both orchards. Trapped males were counted using a stereomicroscope, and counts were transformed to males per trap per day.

Additionally, a mating disruption index (MDI) [35] was calculated after the pheromone application for each monitoring date when controls captured at least 0.4 males × trap^−1^ × day^−1^. This index indicates the percentage reduction of male captures in pheromone traps in disruption plots (MCD, mean of 5 plots) in relation to control plots (MCC, mean of 5 plots), using the following equation:

MDI (%) = [1 − (MCD/MCC)] × 100(1)

### 2.3. Mealybugs Population and Damage

To assess the abundance of *P. calceolariae* population on the plants, every two weeks (spring-summer) or monthly (autumn-winter), twelve plants were randomly selected in each plot and inspected for 3 min counting nymphs, ovisacs, and adult females present.

In season 2017–2018, the damage, i.e., presence of mealybugs on fruits at harvest (April for apples and May for tangerines), was estimated by inspecting 120 fruit per plot (total = 1200 per field), and counting the number of mealybugs present (ovisacs, nymphs, and adult females) and the percentage of infested fruits. In the season 2019–2020, damage at harvest was evaluated in the case of apples (April 2020) by inspecting 50 apples per plot (500 total for the field). Apples were cut and checked under a stereomicroscope observing the presence of individuals inside the fruit (calyx and peduncle).

### 2.4. Statistical Analysis

Male counts (males × trap^−1^ × day^−1^) were analyzed for different periods using Generalized Linear Models (GLM) with Poisson error distribution and log link, with treatment and date as independent variables. When more than two means were compared, Tukey HSD test were used. For periods or dates with very low density and many zero counts, we used non-parametric Kruskal-Wallis ANOVA. The time periods compared were before pheromone application in the first season (September to December 2017), first season after treatment applications (January–April 2018), first season winter (May–July 2018), second growing season (September 2018–April 2019 for apples and only September–December 2018 for tangerines due to the very low counts after that), second season winter (May–July 2019), third growing season after 2nd pheromone application (September 2019–March 2020). We also compared male captures at the beginning of the third growing season, after renewing the dispensers of traps but before the second pheromone application, using GLM (Poisson error distribution and log link) with treatment as the independent variable.

Mealybug population density on plants (mealybugs × plant^−1^) per season were compared by non-parametric Kruskal–Wallis, as populations were always very low or zero on many occasions. Mealybug damage was analyzed only for apples in the 2017–2018 season, using GLM with binomial error distribution and logit link for fruit infestation and Poisson error distribution and log link for insects per fruit. For the 2019–2020 season in apples and for tangerines only a few insects were found on fruits, so no statistical analyses were carried out. Means presented in figures and text are adjusted means of untransformed data ± 1 standard error. Statistical analyses were done with Infostat version 2019 [51] and R 4.0.4 [52].

## 3. Results

### 3.1. Mating Disruption Effect on Males Captures and Persistence in the Apple Orchard

**Figure 1:**
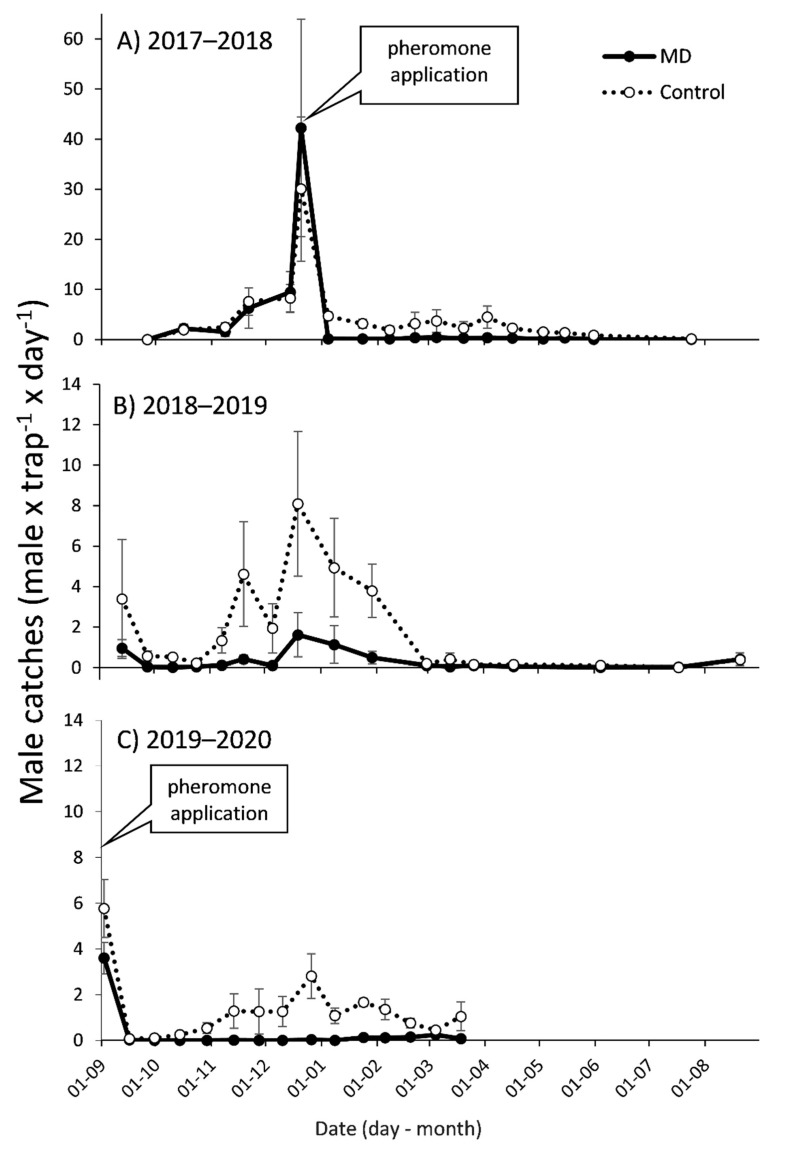
Season long adult male *P. calceolariae* (mean ± SEM) captures in pheromone baited traps in mating disruption (MD) and control plots in apple orchard from 1 September to 31 August of (**A**) 2017–2018, (**B**) 2018–2019 and (**C**) 2019–2020 seasons. Pheromone application dates: 19 December, 2017 and 29 August, 2019. Note that the scale for the Y axis in the first season differs from the rest.

In the first season, before deploying the treatment, male captures were low and increasing in time (Figure 1A). For this period (September–December 2017, 6 monitoring dates) there was no effect of treatment (F_1,48_ = 0.45, *p* = 0.507), with male captures before treatment being similar in the plots that later received the pheromone applications and the control plots (mean captures MD = 4.79 ± 0.52, control = 4.30 ± 0.52 males × trap^−1^ × day^−1^). There was a date effect (F_5,48_ = 108.02, *p* < 0.0001), with the largest male captures right before the treatment applications (Figure 1A). No effect of treatment × date was observed (F_5,48_ = 1.43, *p* = 0.232).

**Figure 2:**
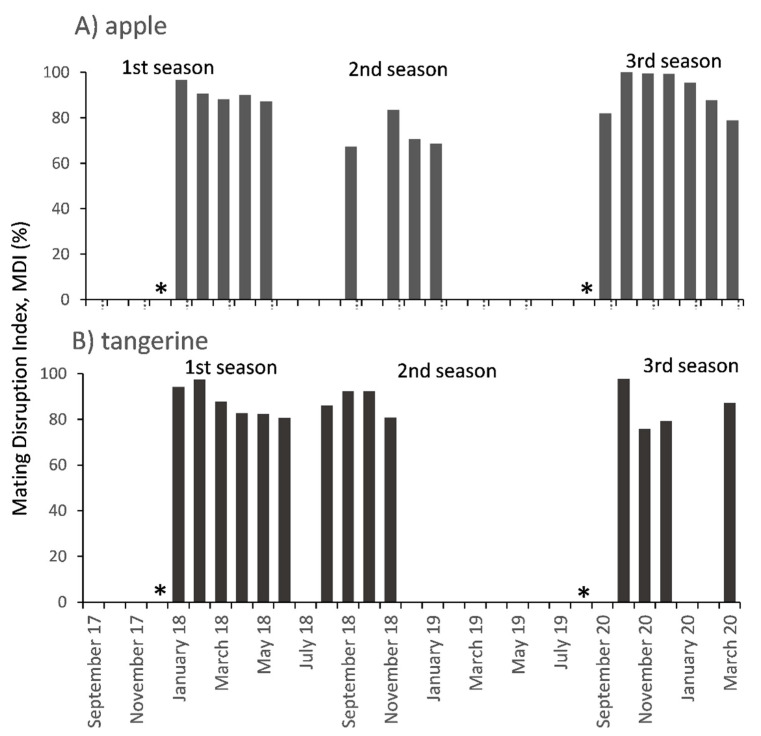
Mating Disruption Index (%), for the three seasons when male captures > 0.4 males × trap^−1^ × day^−1^ for the (**A**) apple and (**B**) tangerine orchards. * Date of pheromone application.

In the rest of the first season (January–April 2018), after deploying the treatments, male captures were lower (Figure 1A). For this period (7 monitoring dates) there was an effect of treatment (F_1,66_ = 45.76, *p* < 0.0001), but no effect of date (F_6,66_ = 0.43, *p* = 0.856) or treatment × date (F_6,66_ = 0.29, *p* = 0.938). Male captures were more than tenfold larger in control than in MD plots, with the latter being very low (“trap shutdown”) (mean January–April: MD = 0.22 ± 0.1, control = 3.06 ± 0.3 males × trap^−1^ × day^−1^). In this period, one month after the pheromone application, MDI was 97% and remained around 90% for the rest of the season (Figure 2A). In autumn–winter of the first season, similar results were observed (May–July 2018, 4 monitoring dates; Figure 1A), with an effect of treatment (F_1,32_ = 5.04, *p* = 0.032), but no effect of date (F_3,32_ = 1.08, *p* = 0.372) or treatment × date (F_3,32_ = 0.24, *p* = 0.865). Male captures were very low, but still sevenfold larger in control than in MD plots (mean May–July: MD = 0.10 ± 0.1, control = 0.70 ± 0.2 males × trap^−1^ × day^−1^).

Although the pheromone was not renewed in the second season, there was a treatment effect (September 2018–April 2019, 14 monitoring dates, F_1,54_ = 9.71, *p* = 0.003). Male captures in MD plots were still significantly lower than in control plots (mean September 2018–April 2019; MD = 0.16 ± 0.1, control 0.98 ± 0.2 males × trap^−1^ × day^−1^, Figure 1B). There was also a date effect (F_13,54_ = 3.18, *p* = 0.001) but not a treatment × date effect (F_13,54_ = 0.21, *p* = 0.998). Male captures were larger in December, similar to the first season, and lower in October and April, although not significantly different (Figure 1B). The mean of MDI for this season was 73% (Figure 2A). During winter of the second season (June–July 2019, 2 monitoring dates) captures in both MD and control plots were very low and similar (mean captures: MD = 0.02 ± 0.1 and Control 0.04 ± 0.1 males × trap^−1^ × day^−1^, Figure 1B). No effects of treatment, date or treatment × date were found (*p* > 0.8 for all).

At the end of August 2019, before the beginning of 2019–2020 season, after renewing the lures in the traps (20 August) but before the new pheromone applications (2 September), there was an increase in male captures, being similar for MD and control plots (MD = 3.60 ± 0.9, control = 5.77 ± 1.1 males × trap^−1^ × day^−1^; F_1,8_ = 2.47, *p* = 0.16; Figure 1C). Subsequently, after the second pheromone application in this third season, male captures in the MD plots were very low (0.0 ± 0.0 males × trap^−1^ × day^−1^) while in the control plots, even when captures were also low (0.68 ± 0.1 males × trap^−1^ × day^−1^), they followed the temporal dynamics observed in previous seasons, with a peak by the end of December (Figure 1C). Significant reduction of males captures in MD plots compared to control plots was detected for October and then from December through February (H > 4.36; *p* < 0.05 for all). Mean MDI for this period was 92% (Figure 2A).

### 3.2. Mating Disruption Effect on Males Captures and Persistence in the Tangerine Orchard

**Figure 3:**
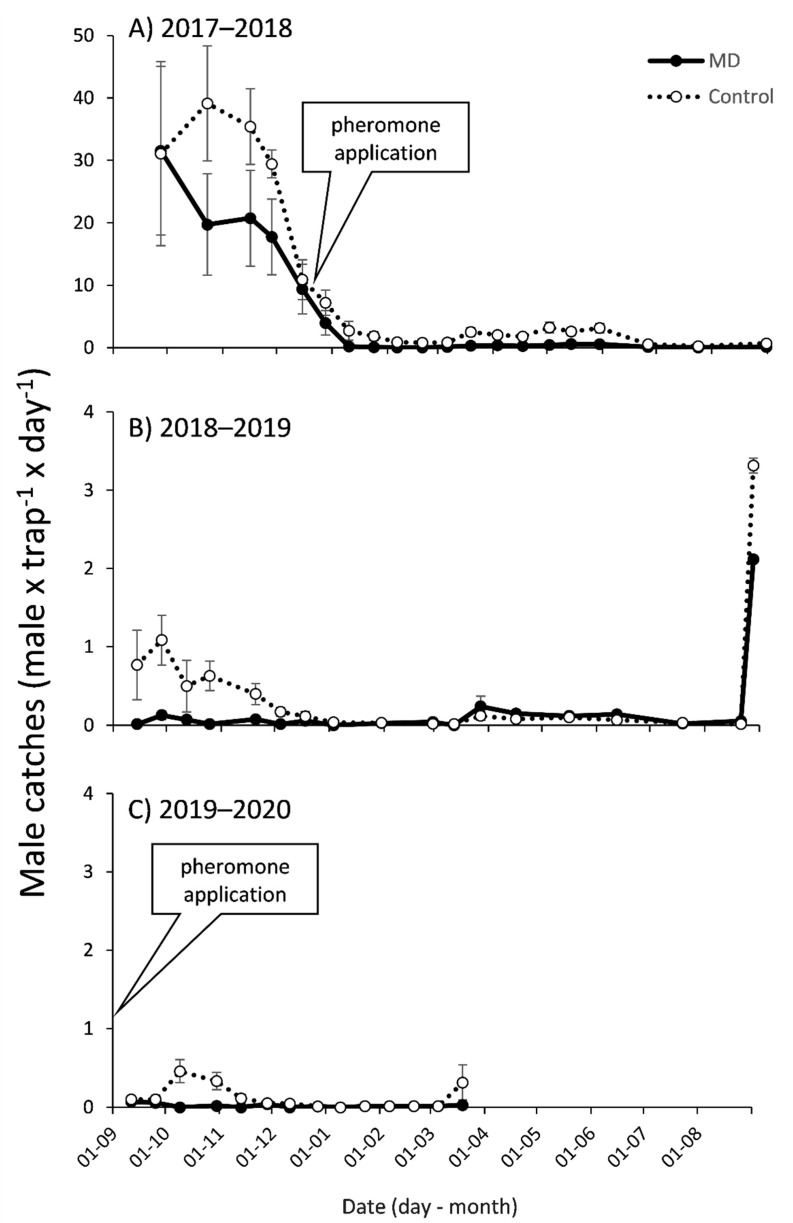
Season long adult male *P. calceolariae* (mean ± SEM) captures in pheromone baited traps in mating disruption (MD) and control plots in tangerine orchard from 1 September to 31 August of (**A**) 2017–2018, (**B**) 2018–2019 and (**C**) 2019–2020 seasons. Pheromone application dates: 20 December 2017 and 2 September 2019. Note that the scale for the Y axis in the first season differs from the rest.

In the first season, before deploying the treatments, male captures were high but decreasing in time (Figure 3A). For this period (5 monitoring dates) there was an effect of treatment (F_1,39_ = 33.65, *p* < 0.0001), date (F_4,39_ = 26.16, *p* < 0.0001) and treatment × date (F_4,39_ = 5.52, *p* = 0.001). Plots that would receive the control treatment had larger populations than those that would receive the MD (mean captures September–November 2017 MD = 18.5 ± 0.9; control = 26.8 ± 1.1 males × trap^−1^ × day^−1^). Nevertheless, for the date previous to the treatment applications, male captures were lower and similar for both (MD = 9.4 ± 1.4; control = 10.9 ± 1.5 males × trap^−1^ × day^−1^, LSD *p* > 0.05). Male captures significantly decreased in this period, with the least numbers in the last date.

During the rest of the first season (December 2017–April 2018), after deploying the treatments, male captures were lower (Figure 3A). For this period (9 monitoring dates) there was an effect of treatment (F_1,70_ = 16.24, *p* = 0.0001), date (F_8,70_ = 9.51, *p* < 0.0001) but not for treatment × date (F_8,70_ = 1.41, *p* = 0.206). Male captures were tenfold larger in control than in MD plots, with the latter being very low (“trap shutdown”) (mean December–April: MD = 0.17 ± 0.1, control = 1.78 ± 0.2 males × trap^−1^ × day^−1^). Male captures were also significantly larger in the at the beginning of this period than the rest of the dates. In this period, one month after the pheromone application, MDI was 94% and then remained > 80% (Figure 2B). Similar results were observed for the fall-winter of the first season (May–August 2018, 6 monitoring dates; Figure 3A) with an effect of treatment (F_1,48_ = 10.03, *p* = 0.003), date (F_5,48_ = 2.43, *p* = 0.049) but not for treatment × date (F_5,48_ = 0.07, *p* = 0.997). Male captures were fivefold larger in control than in MD plots, with the latter being very low (mean May–August: MD = 0.20 ± 0.1, control = 1.15 ± 0.3 males × trap^−1^ × day^−1^). MDI in this period was also above 80% (Figure 2B).

In the second season (2018–2019) when pheromone application was not renewed but dispensers were left on the trees, male captures in the pheromone traps were much lower than the previous season, and mostly concentrated in the spring (Figure 3B, note the change in scales of Y axis). In this period (September–December 2018, 7 monitoring dates) an effect of treatment (F_1,56_ = 4.93, *p* = 0.031), but not for date (F_6,56_ = 0.39, *p* = 0.885) or treatment × date (F_6,56_ = 0.15, *p* = 0.989) was observed. Male captures were very low, but nevertheless they were tenfold larger in control than in MD plots (mean September–December: MD = 0.04 ± 0.04, control = 0.41 ± 0.13 males × trap^−1^ × day^−1^). During fall and winter (May–August 2019, 4 monitoring dates) captures in both, MD and control plots, were low and similar (mean May–August: MD = 0.07 ± 0.07, control = 0.04 ± 0.06 males × trap^−1^ × day^−1^) (Figure 3B). No effects of treatment, date or treatment × date were found (*p* > 0.8 for all).

At the end of August 2019, before the beginning of 2019–2020 season, after renewing the lures in the traps (21 August) but before the new pheromone applications (29 August), there was an increase in male captures, being similar for MD and control plots (MD = 2.11 ± 0.7, control = 3.32 ± 0.8 males × trap^−1^ × day^−1^; F_1,8_ = 1.31, *p* = 0.29; Figure 3B). After that, male captures were low, with a small increase in captures in the control plots in October 2019 and March 2020 (Figure 3C). In this period (September 2019–March 2020, 14 monitoring dates), MD plots had significantly fewer male captures than control plots (H = 40.94; *p* = 0.0005; Figure 3C). For October 2019, when the small peak in male captures in the control plots was observed, MDI was 98% (Figure 2B).

### 3.3. Mealybugs Population and Fruit Damage in Tangerines and Apples

During the whole evaluation period, mealybug population levels on the plants (visual counts) were exceptionally low in both orchards, with no significant differences between MD and control for all seasons (Table 1).

In the first season, the number of mealybugs and percentage of infested fruit was very low in both crops. In apples, fruit infestation was 4 ± 1% in MD plots and 2 ± 1% in control plots, without significant differences (F_1,8_ = 3.10, *p* = 0.12). Number of *P. calceolariae* per fruit were also similar and low (MD = 0.06 ± 0.11, control 0.05 ± 0.10, H = 0.39, *p* = 0.59). In the 2019–2020 season, out of the 500 apples harvested and inspected, only one presented two *P. calceolariae* nymphs.

In tangerines, only 4 fruits (out of the 1200 inspected) of the control plots were infested, with a total of 10 *P. calceolariae* individuals. This resulted in a 0.67% fruit infestation in the control plots and 0% infestation in the MD plots. Interestingly, a 9% fruit infestation with *P. longispinus* was found in both control and MD plots.

## 4. Discussion

The results of our experiments provide valuable information about the potential of mating disruption for the control of *P. calceolariae* in two fruit crops of worldwide economic importance, such as apples and tangerines. Previously, studies showing the usefulness of this technique as a control tool for a mealybug species have been carried out only for *Pl. ficus* in grapes [15,34,35,37,38], leading to commercial formulations. Our results showed that in MD plots “trap shutdown” occurred, as male captures in pheromone traps were reduced by 97% to 100% in both fruit crops one to two months after the applications in two seasons.

The pheromone doses used in this study (6.32 and 9.45 g/ha of the stereoisomeric pheromone mixture) were much lower than those used in studies with *Pl. ficus*, which found effects with 62.5 and 93.8 g/ha [35]; 93 g/ha [36]; 54 to 90 g/ha [38] and 20.0 to 61.7 g/ha [15]. When lower doses were tested, such as 4.15 and 8.3 g/ha, male trap captures were similar in the MD and control treatment [15]. In our study with *P. calceolariae*, with ten times less pheromone than other studies with *Pl. ficus*, we obtained MDI between 80 and 100% in both crops and seasons. In addition to the dosage, the type of dispenser may play an important role in the successful implementation of mating disruption. In previous MD studies with *Pl. ficus*, other different types of dispensers and formulations have been used, including Checkmate VBM-XL (Suterra LLC., Bend, OR, USA), Isonet^®^ PF (Shin-Etsu Chemical Co.Ltd., Tokyo, Japan) and a sprayable microencapsulated formulation [15,35,36,37,38]. Our study reports for the first time the use of SPLAT^®^ as a dispenser for MD of mealybugs. The SPLAT^®^ technology for mating disruption has been used and commercialized mainly in Lepidoptera [45]. The results presented here demonstrate the feasibility of using SPLAT^®^ as a pheromone dispenser for MD in mealybugs.

Interestingly, MDIs calculated during the first season indicated that the mating disruption effect was maintained at least 12 months after the application of the pheromone treatment, with mean MDI values between 65 and 80%. Moreover, in the following season, 2018–2019, when no pheromone was applied (but dispensers were left in the orchard), MDI was around 80% during spring in tangerines (August to November, 2018) and during spring and summer (September 2018 to January 2019) in apples before trap captures declined. Low male captures were observed in both MD and control plots during the autumn-winter months (2018 and 2019), since there is a natural decrease of the populations due to the lower temperatures of the winter months. The amount of pheromone released or remaining in dispensers (SPLAT^®^) over time in the field was not measured in our study, so the results observed in the second season could be due to residual emission from the dispensers left from the previous season, because of the carry over effect of lower populations or some other factor. Therefore, to understand more comprehensively the eventual persistence of a MD pheromone release effect from dispensers under different conditions, it should be measured systematically through time. This is important, because for successful population control the pheromone not only should remain active for a long period in the field, but it is also key that it be consistently released in a constant amount [38]. The previous studies with *Pl. ficus* in grapes only evaluated the MD treatments up to six or seven months, maintaining a satisfactory disruptive effect in males [15,35,37]. However, in evergreen fruit trees such as tangerines, the MD effect needs to be maintained for a longer period of time to cover the productive period fully. Our results show that during the second season 2018–2019 (without pheromone renewal), the mean mating disruption index for the months of September to November was 88% in tangerines, when a male flight peak occurs. This long-lasting treatment effect, 6 to 12 months, in mealybug MD is desirable for farmers, because it makes this technique cost effective compared to insecticide applications. Pheromones can also be used in combination with insecticides or biological control, potentially generating a synergistic effect on mealybug control [15].

Trap shutdown is one of the main signs that mating disruption is working [35], but to measure control effectiveness it is desirable to demonstrate that populations and damage are reduced [2,46]. The abundance of mealybugs was extremely low throughout the duration of the trials in both crops in our study. Due to this low abundance, we were not able to observe a reduction of populations on trees or even of damage of fruits. Probably the pest control measures taken by the farmers in the experimental fields also impacted mealybug populations on the plants and did not allow us to evaluate properly the impact of MD through visual monitoring and fruit infestation. It is important to note that studies have shown that MD is more effective when used at low population levels [35,37,38], while at high mealybug levels at least two continuous seasons will be needed to produce an observable effect of MD [36], or it might even be necessary to combine the use of MD with insecticide applications [37]. Another factor to consider when dealing with high mealybug populations is that high doses of pheromone are needed, as otherwise the disruptive effect can decrease due to the high abundance of females as natural sources of pheromone [15]. Thus, studies in orchards with larger populations of *P. calceolariae* are desirable to test the potential of MD for control in a wider range of field situations.

This study shows that mating disruption has a good potential for controlling *P. calceolariae* in fruit crops, as previously demonstrated for *Pl. ficus* in grapes [34,37,38]. The final growers’ decision to implement disruption will depend on several factors, such as the cost of the pheromone product, efficiency of the technique, pest population level and cost comparative to other control alternatives available (e.g. biological control, insecticides) [53]. Nevertheless, it is important to consider that in quarantine pests very low populations are required to avoid infestation of the products before harvest, and that MD is a more efficient technique at lower population levels. Moreover, it has been found that a low pheromone concentration is sufficient to provide good control at low pest population levels [54]. The combination of higher pheromone concentration and insecticide application may be necessary with high pest populations, which may increase the cost of implementing the technique, and thus not be economically sustainable [53,54]. However, the long-lasting effect of the pheromone would make it cost-effective compared to repetitive insecticide applications. The use of this pheromone-based technique instead of insecticides also has the advantage of being environmentally friendly and non-toxic.

## Figures and Tables

**Table 1:** Mean number of mealybugs (all life stages) per plant over the season for the three seasons studies, in mating disruption (MD) and control plots in tangerine and apple orchards. Twelve plants per experimental unit were monitored (60 plants per treatment in each date).

Fruit Crop and Season	Treatments		Kruskal-Wallis Test
	MD	Control	
	Mealybugs × Plant^−1^ ± SE		
**Apple**			
September 2017–April 2018	0.27 ± 0.09	0.04 ± 0.01	H = 0.28; p = 0.59
September 2018–April 2019	0.59 ± 0.14	0.30 ± 0.08	H = 1.47; *p* = 0.22
September 2019–March 2020	0.03 ± 0.01	0.13 ± 0.04	H = 2.79; *p* = 0.09
**Tangerine**			
September 2017–April 2018	0.30 ± 0.08	0.31 ± 0.09	H * = 0.0; p = 0.96
September 2018–April 2019	0.02 ± 0.009	0.001 ± 0.001	H = 3.83; *p* = 0.05
September 2019–March 2020	0.00 ± 0.0	0.00 ± 0.0	NA *

* not applicable.

## Data Availability

The data presented in this study are available on request from the corresponding author.
